# Aging in Context: Incorporating Everyday Experiences Into the Study of Subjective Age

**DOI:** 10.3389/fpsyt.2021.633234

**Published:** 2021-04-09

**Authors:** Matthew L. Hughes, Dayna R. Touron

**Affiliations:** Department of Psychology, The University of North Carolina at Greensboro, Greensboro, NC, United States

**Keywords:** subjective age, situational context, multidimensionality, health behavior, metacognition, self-awareness

## Abstract

The age that a person feels is a strong predictor of their well-being and long-term health, beyond chronological age, showing that people have a self-awareness that provides insight into their aging process. It appears this insight has broad implications for a person's everyday life and functioning. One's subjective age is shaped by metacognitive beliefs about aging, including both expectations about typical changes but most notably the awareness and interpretation of personal experiences. Subjective age has been described as multidimensional, aligning with life domains such as cognitive, social, and physical functioning. This perspective, coupled with laboratory studies that manipulate subjective age, suggests that situational context has an important role in determining the age a person feels. Here we review literature on subjective age with a focus on how research and theoretical perspectives should be adapted to integrate momentary experiences. We propose a contextual model that will help discriminate the links between situational influences and subjective age, as well as resulting behaviors that impact health and well-being. While most research has considered subjective age to be a relatively stable variable, we provide a novel account of how daily life offers a variety of situational contexts and experiences that directly impact the age a person feels at a given moment. We propose that studying moment-to-moment context is a critical next step in understanding the associations between subjective age, lifestyle choices, and health outcomes.

## Introduction

The aging process has you firmly in its grasp if you never get the urge to throw a snowball

–Doug Larson

Age is, at its most simple, a measurement of time since birth. But we sometimes consider age as less inevitable, as a state of mind that is malleable and idiosyncratic. Researchers have found that older adults typically feel 20% younger than their actual age (1), in contrast to young adults who tend to feel older. In younger adults, an older subjective age seems to reflect a feeling of maturity. For example, one study measured subjective age in a sample of adolescent participants over a 2 year span (2). They found that experience with more “adult” activities, such as sex or substance abuse, was associated with an older subjective age over the 2 year span. The trend of young adults reporting an older subjective age continues until about age 25 (3, 4), when people begin to report feeling younger than their actual age. This discrepancy grows larger until the 60s, when it becomes more stable.

Although subjective age has been studied for many years, we argue in this paper that much is still unknown about the specific experiences that lead people to feel younger or older than their lived years, and how these feelings of self-awareness impact health and wellness. People encounter dozens of contexts each day that may affect how old they feel, and older adults are particularly likely to encounter contexts that make their age highly salient. These age-salient contexts may cause people to reflect on, and change, how old they feel. Fluctuations in felt age may impact lifestyle choices in the moment, which may help explain how subjective age predicts long-term health and well-being outcomes.

Subjective age has been conceptualized as both an outcome of various personal factors and also as a causal influence. A major focus in this research area has been understanding the influence of subjective age on important life outcomes. For example, Westerhof and Wurm (5) proposed a theoretical model that described subjective age influencing psychological resources, such as metacognitive and general control beliefs, will to live, and health behaviors. These psychological resources were linked with health, which in turn was linked with survival.

Perspectives on subjective age have generally acknowledged, either explicitly or implicitly, that situational context is a component of how old a person feels; in one context a person may feel older, while in a separate context that same person may feel younger. Subjective age is made up of multiple dimensions from important domains from daily life [e.g., (6, 7)]. However, none of this work to date accounts for an impact of momentary contextual shifts on subjective age. In this paper, we will argue why and how research on subjective age should adopt novel approaches to assess moment-to-moment subjective age and the fluctuation in felt age that occurs across everyday situations.

## Proposing a Contextual Model Subjective Age

**Figure 1:**
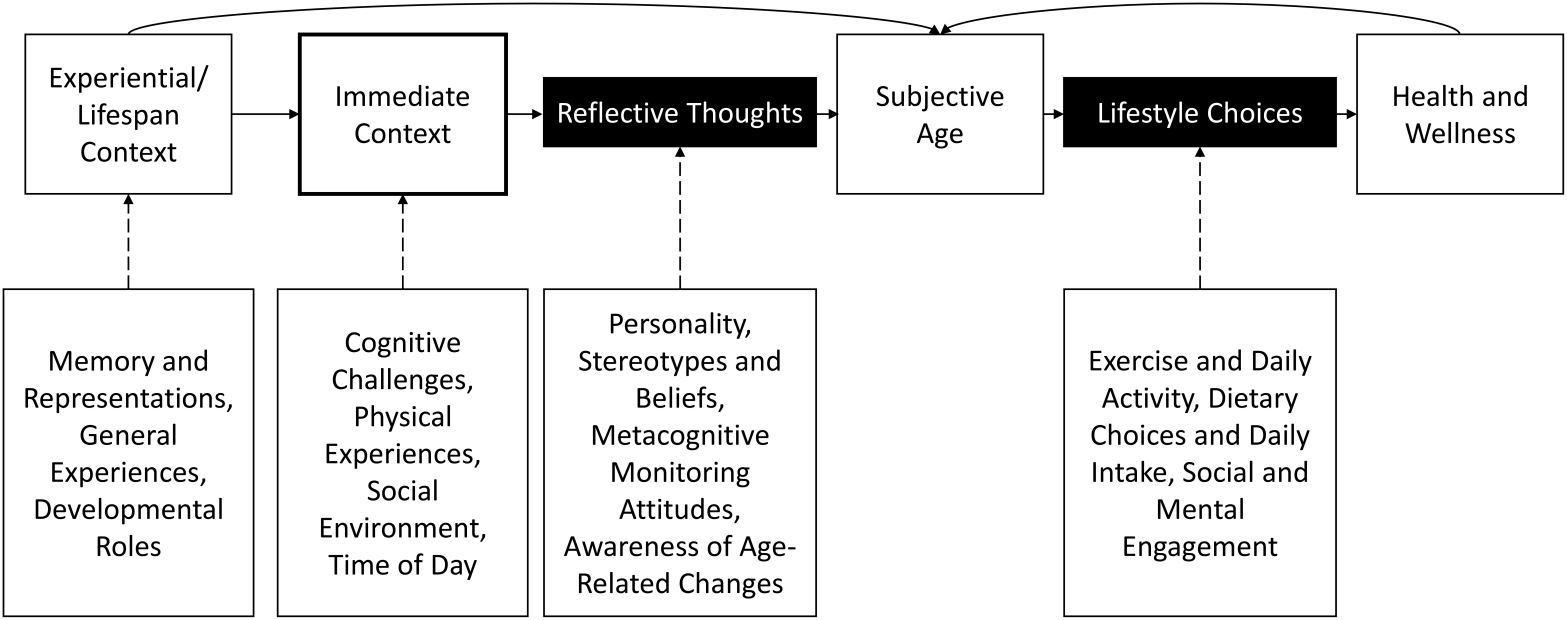
Contextual model of subjective age. Subjective age is influenced by context through reflective thoughts.

In this paper we introduce a **contextual model of subjective age** that formally incorporates the momentary situational factors that make us feel a certain age, and how our resulting behaviors have consequences for health and well-being (Figure 1). We believe that subjective age research will benefit from a unification of these perspectives; our contextual model of subjective age is a first step toward that aim. Similar to the model by Westerhof and Wurm (5), our model stresses the relevance of individual experiences and perceptions for subjective age. It is important to note that the model we provide here represents defined space within a complex system. We agree that other factors, such as social roles, aging expectations, and awareness of age-related changes, form a broader frame for understanding the precursors and outcomes of subjective age. For example, Diehl et al. (8) described how self-knowledge, such as subjective age and self-perceptions of aging, contributes to a person's Awareness of Aging. In their model, subjective age is rooted in personal experience, and expressed consciously when the proper context is provided. Self-perception of aging is a form of self-knowledge that is especially relevant in midlife or older adulthood (8).

Our model expands past work as we address and incorporate the issue of how one's immediate and proximal **situational context** impacts subjective age. Such contextual influences represent a gap in the current subjective age literature, and may include information from the environment, such as location or the presence of peers, or intrinsic experiences, such as reflections on experiences of difficulty or success. For example, do older adults feel subjectively younger in the presence of same-age peers, or subjectively older around children? There is evidence that older adults distance themselves from older age identities (9), but little is known about the influence of others on subjective age. Our proposed model includes these factors for confirmatory studies.

Prior models of subjective age have proposed considering a person's lifetime of experiences. This life-span developmental approach links subjective age with health by focusing on key constructs such as age stereotypes, personality, and self-perceptions of aging (5, 10). As an illustration of the link between immediate context and subjective age, our model allows that an individual may feel older in situations when they are challenged, and younger when they are successful. These experiences may most directly impact subjective age when people perceive and reflect on them as relevant to their own aging process. Such contexts might also lead to reflective thoughts that indirectly impact subjective age, such as the priming of ageist stereotypes. As an important note, we are not suggesting that daily reports of subjective age are unreliable; on the contrary, there exists a large body of research that people's daily assessments of subjective age are reliable (11). Rather, we propose that subjective age fluctuates in meaningful ways in response to one's momentary context.

As the next link in the model, felt age influences one's active lifestyle choices. For example, when one feels older, they may be less likely to take their daily walk or attend a social gathering. Contextual influences likely play a crucial role at this step as well, from the availability of certain lifestyle opportunities to access to healthcare. Gaps in the literature also must be addressed at this step. The last step of the model describes outcomes on cognition, health, and well-being as immediate and cumulative effects of these lifestyle choices. Here we clearly see ties to the existing literature that find that subjective age is associated with health (12, 13), cognition (14), and even mortality (15). Our model helps to specify and explain the intermediary steps that link subjective age with those well-being outcomes.

Our contextual model of subjective age incorporates recent research findings as well as earlier theoretical perspectives. Hubley and Hultsch (16) described a reciprocal relationship between self and society. This view fits well within our model; society can serve as a broad situational context acting upon subjective age by affecting how an older adult sees their self-relative to society, and will also influence their lifestyle choices by presenting opportunities and challenges to daily activities. Several studies have demonstrated that subjective age reflects life experience [see (17) for a review], and we agree. However, this broad consideration of life experience, though important, neglects the impact of specific everyday experiences such as grocery shopping, walking a dog, or going to a movie, on subjective age and its proximal outcomes. Our model incorporates the immediate situational context as a key factor in determining momentary subjective age.

In the next sections of this paper, we review research findings and influential theoretical perspectives related to the impact of situational context on subjective age. We then look at how we currently study subjective age and address how these approaches do not precisely account for situational context. Finally, we argue for new methodological approaches that can harness contextual variation to help us better understand the nature of subjective age.

## Constructing Subjective Age

Although a wide range of research examines the correlates of subjective age, there is relatively little research into the factors that people actively consider while constructing subjective age. In one effort, Teuscher (18) models subjective age using Kastenbaum et al.'s (6) original multidimensional framework. Participants were asked to report their subjective age first broadly, then on eight specific domains as compared to “people your own age.” These domains were: body fitness, activities, relation to other people, interests, mental abilities, esteem by society, esteem by family and friends, or attractiveness. Each answer could range on a five-item scale from “much older” to “much younger.” These nine subjective ages (general and domain specific) loaded on to two component factors, one which described social and mental subjective ages, and one which focused on physical subjective ages. These findings highlighted that physical and health factors affect subjective age separately from mental factors.

Montepare (19) proposed that subjective age is influenced both by distal reference points, such as aging expectations or past experiences, and proximal reference points, described as personal events that serve as age markers. Proximal reference points can be classified as historic, such as birthdays and anniversaries; physical or health related, such as strokes and memory loss; normative, such as retirement; or interpersonal, such as interactions with another person who differs in age. Distal reference points serve as anchors for subjective age and are responsible for subject age change over the lifespan, whereas proximal reference points are responsible for within-person fluctuation in subjective age. In the absence of relevant proximal reference points, distal references assert more influence. As additional proximal references are added, one's subjective age is updated.

The life-span perspective of subjective age included a process of updating personal age perceptions throughout the life course (19–22). Major life events, such as retirement, widowhood, or becoming a grandparent, serve as markers that make aging salient, while health-related events such as heart attack, stroke, or menopause provide cues as to how well one is aging. For example, studies have found that older adults' felt age was closer to their chronological age near their own birthday but was younger when their birthday was more distant (7), and that people who became grandparents earlier in life than expected felt subjectively older than those who became grandparents later (23). This highlights the importance of immediate context for subjective age in our model.

Further developing this lifespan framework, Barrett and Montepare (2015) apply five life course principles to subjective age. These include: a person's life-long development, their agency in making their own decisions, the historical time and place in which they live, the timing of events and consequences in their life, and the idea that their life is linked with other individuals in their social network. Although many of the principles in this framework point to changes that occur across the macro-level of the lifespan, many of these same principles can work on a micro-level basis as well. For example, instead of looking at the geographical place a person lives in, we can look at the types of environments a person experiences throughout the day. We can use the principle of agency to examine the types of momentary decisions a person makes, and how the timing of these events impacts subjective age throughout the day.

In our contextual model of subjective age (Figure 1), the age one feels is impacted by their response to experiences that occur in everyday life. Accordingly, it is critical that we not only measure everyday life contexts using a method such as experience sampling, but also measure the thoughts and reflections people have when constructing their subjective age. In the model, this is depicted by the link between immediate context and subjective age passing through a box representing an individual's reflective thoughts.

As noted above, it is also important to recognize the distinct dimensions of subjective age by asking participants to report their overall subjective age as well as how old they feel within specific life domains. People assess their age-related changes using factors such as their physical health, mental performance, and social activities (24), and take these factors into consideration when constructing their subjective age. Therefore, a challenge in one of these particular areas, such as a “tip-of-the-tongue” memory failure, might lead to an increase in cognitive subjective age, but not the other dimensions such as physical subjective age, similar to how stereotype embodiment theory works on multiple pathways (25). Likewise, cognitive success, such as knowing the answer to a trivia question on the radio, might lead to a younger cognitive subjective age. Future research should focus on these considerations and interventions that may target specific domains.

This is some evidence for a link between subjective age and everyday behaviors. Montepare (26) explored the relationships of several domains of subjective age (Felt Age, Psychological Age, Physical Age, Social Age, Age Identity, and Age Awareness). Younger subjective age predicted more exercise behaviors such as taking walks or going to the gym, grooming behaviors such as flossing or getting a manicure, and social behaviors such as taking vacations or interacting with younger individuals. Age awareness, conversely, was negatively associated with medical behaviors such as seeing a doctor or preparing for retirement. This suggests that the age people felt plays a role in their everyday behavior. These results were correlational, so it was impossible to determine whether subjective age impacted behavior, or behaviors informed people's felt age.

## Outcomes and Predictors of Subjective Age

One of the primary reasons for the continued interest in subjective age is its value as a predictor of well-being and long-term health outcomes. Older subjective age predicts poorer future health (12, 13), cognitive decline (14), increased hospitalization risk (27), morbidity (28), nearness to death (29), and mortality (15), above and beyond chronological age. Subjective age also predicts future physical limitations (30). The English Longitudinal Study of Ageing (ELSA) asked people how old they at two waves 4 years apart. They also asked people about any difficulties they had with activities of daily living (ADL). Subjective age accounted for 7% of the variance in ADLs 4 years later; participants who felt older at the baseline wave reported greater difficulty with ADLs at the next wave.

Subjective age is also tied to biomarkers of aging. Older subjective age is accompanied by higher levels of cystatin C, a marker of kidney function (31) and neurological measures that indicate an older brain age (32). Participants who reported feeling younger than their actual age had larger gray matter volumes in the inferior frontal gyrus and superior temporal gyrus, compared to participants that reported feeling their age or feeling older than their age. Degradation in these areas may affect how a person perceives age-related changes and lead to cognitive decline, such as a reduction in inhibitory control, which may affect how people assess and react to daily life events. Kwak et al. note that their analytic models assumed that subjective age is a stable variable that accurately reflects the aging process, particularly of the brain.

In addition to subjective age predicting important aspects of well-being, the converse is also true—various factors predict subjective age. As expected, subjective age increases with chronological age, although subjective age typically lags behind (i.e., is younger than) chronological age [e.g., (33, 34)]. Higher education and better health also predict a younger subjective age [(1, 20); but also see (4, 35)]. While some studies have shown subjective age predicts ADL and IADL (instrumental activities of daily living), this relationship appears to be bidirectional. One study found that better physical functioning among the oldest-old was associated with not feeling old (36). This study used a sample of participants that were all older than 84 and modeled ADL/IADL as a predictor of subjective age. Subjective age was defined by a single question, “Do you feel old?” Participants could respond yes, partly, or no. Participants who reported fewer difficulties on the ADL and IADL were less likely to endorse feeling old. Liang (37) found similar results in a Chinese sample of the oldest-old. Using the same definition of subjective age, Liang found that for a 1 unit increase in ADL/IADL scores, participants were 8% less likely to report feeling old. Other studies have shown small effects of employment status (1) and income (4) on subjective age. We believe that the key to understanding subjective age may not only lie in demographic factors, but also in life experiences and their context.

Another important factor in the link between one's felt age and health might be the types of relationships a person forms and keeps. One study looked at whether a person's marital status would alter the impact of subjective age on well-being (38). Specifically, they were interested in the *quality* of the relationship, whether strong relationships boosted the effect of younger subjective age. Because other work had not found evidence that married people feel any younger or older than unmarried people (14, 39), this study only considered people who were married or in similar long-term relationships using a sample from the Midlife in the Unites Statues (MIDUS) Longitudinal dataset. Using data from the first two waves of MIDUS 10 years apart, this study found that people who feel subjectively younger and who reported higher quality relationships showed higher memory scores and resting heart rate variability, a measure of cardiovascular health. These results suggest that a person's social relationships may impact subjective age and strengthen its effects.

The bulk of subjective age research has been focused on the correlates of subjective age. While that line of work is indeed important, it has not explained the mental process of constructing one's subjective age, nor how a person's experience models it. We attempt to rectify this with our proposed model.

## Theoretical Perspectives: Subjective Age as Self-Knowledge

One of the earliest references to subjective age comes from Kastenbaum et al. (6), whose concept of “personal age” described a combination of how old an individual looks and feels. Their study demonstrated that subjective or personal age was entirely distinct from chronological age, and “that they cannot be interchanged without the likelihood of gross error (p. 205).” Similarly, the concept of age identification (40) represented whether or not an older adult would classify themselves as old. This work noted that people did not identify as old strictly on the basis of their age, and that one of the strongest predictors of an old age identification was whether a person was retired. Age identification research did not produce a felt age represented as a number in years, but rather a categorical response. Even the early work on subjective age only produced responses such as “much younger” or “somewhat older” [e.g., (6)].

Beliefs and feelings about aging may help to explain why older adults report feeling younger than their actual age. Consistent with this view, Montepare and Lachman (41) demonstrated that the discrepancy between subjective age and chronological age was associated with fears of aging and life satisfaction. Older subjective age was associated with less fear of aging and greater life satisfaction, suggesting that people more comfortable with their status in life are more likely to report a subjective age near their chronological age. Stephan et al. (12) found that younger subjective age was positively related to subjective assessments of health and memory self-efficacy, which were also positively related to life satisfaction. These patterns underscore the role of self-assessment and self-knowledge in subjective age and well-being.

The relationship between subjective age and quality of life deserves a deeper look. Although Montepare and Lachman (41) found older subjective age predicted greater life satisfaction, the findings of Stephan et al. (12) suggested the opposite. One explanation for these discrepant results might be that the measurement of subjective age used by Montepare and Lachman was an average of four domains; Feel Age, Look Age, Do Age, and Desired Age. Other work (42) that has used a similar subjective age questionnaire to Montepare and Lachman but separated Desired Age from the other domains support the findings of Stephan et al. (12). This study found that people who reported a younger subjective age also reported greater satisfaction with their age, and this effect was stronger in women than in men (42). Another study suggested that one's aging attitudes explain why feeling older was associated with lower life satisfaction (43). People with positive aging attitudes showed no relationship between subjective age and life satisfaction, but people with negative attitudes showed lower life satisfaction with older subjective age. Taken together, all these studies do suggest a relationship between subjective age and a person's perceived quality of life. When one's desired age is treated separately, people report feeling younger when they are more satisfied with their life. This not only supports the idea that context is important for subjective age, but also that the lifetime experience can influence how old you feel.

Other research supports the view that older adults may dissociate themselves from “other old people,” even to the extent that they reject their own status as an older adult (9). This is theorized as a protective mechanism. One may feel that by denying their status as an older adult, they will not fall prey to the stereotypes of aging. Weiss and Lang found that older adults who weakly identified as older reported feeling like they have more time left, an expanded future time perspective, compared to those more strongly identified as older. Negative age stereotypes may make older adults more likely to dissociate themselves from old age (9). Although this denial may be protective in some ways, the consequences could also be harmful. Rejecting one's age may have implications for health if the person does not recognize and respond to age-related changes. There may also be lasting psychological harm from rejecting an aspect of your identity.

Older adults are aware that their time is finite (44, 45) and may use their perspective on future time to focus and prune social networks and focus on goals related to emotion regulation and satisfaction [Socioemotional Selectivity Theory (46)]. Maintaining a younger subjective age may “stretch” this perceived time out. Although this may help explain why some people may want to maintain a younger subjective age, it does not explain how subjective age predicts health outcomes. For example, wanting to be younger does not readily explain the fact that people with a younger subjective age do live longer (29). One explanation is that people who report a younger subjective age also engage in more healthy lifestyle activities, as we depict in our model (Figure 1).

Because it depends on introspection, subjective age has been described as a form of self-knowledge (17) that demonstrates an understanding of how well one is aging. Each of the perspectives described above relies on the general notion that personal knowledge and beliefs impact subjective age. Yet researchers have studied subjective age for years without directly capitalizing on the fact that a person must engage in reflective thoughts when considering how old they feel. Discovering the situational factors and resulting thoughts that people use to construct their subjective age will open new avenues of research. With our contextual model, we argue that we must understand what people consider while reporting their subjective age in order to focus future research on the immediate factors and situations that make a person feel older or younger. This targeted approach may also enable the development of interventions that can use the self-knowledge of subjective age to improve lifestyle choices and, by extension, health and well-being. For example, if we discover situations that make adults feel older and also less likely to engage in exercise, we could focus our research on ways to alleviate that elevated subjective age to promote exercise engagement.

## Situational Influences on Subjective Age

Efforts to understand the dynamic nature of subjective age as a form of self-assessment has received an increased focus. Research has begun to consider not only the predictive power of subjective age, but also how subjective age operates. In our model, we propose that subjective age is an internal process; people engage in thoughts about their performance and assess how old that performance makes them feel. There is some evidence that supports this. Several studies have shown subjective age as malleable and susceptible to influence from external factors. Eibach et al. (47) induced an older subjective age by having older adults read a blurry passage of text. Participants were either told that the blurry passage was the product of a printing error or given no explanation. Participants who received no information reported an older subjective age than those told of the printing error, presumably because they internalized the blurry text as a sign of age-related visual decline.

Other work with physical self-assessments has extended the finding that external factors and feelings of success can immediately influence subjective age. In one study, participants were given a grip strength test and then given feedback on their performance (48). Half of the participants were told their grip strength was higher than 80% of their peers, while the other half received no feedback. Those who received the positive feedback reported a younger subjective age than those in the no feedback group. Those who received the feedback also demonstrated an improvement in their performance on a second grip strength test. This finding suggests that not only can feedback impact subjective age, but that this impact may also have implications for later performance. More work is needed to determine whether the positive feedback alone would lead to an improvement on a second grip strength test, or whether the change in subjective age might play a mediating role. Another possibility is that the feedback was particularly impactful because it contradicted the participants' expectations. Older adults may expect that their grip strength has declined over the years, so feedback that contradicts this may have provided a “boost” to their subjective age, making them feel younger. Although these studies were conducted in a laboratory, they support the contention that specific momentary experiences and challenges, such as those encountered in daily life, drive fluctuations in subjective age.

Among these daily experiences are physical challenges and reminders of body age that are also important for subjective age (49). Using longitudinal data from the National Health and Aging Trends Study, Barrett and Gumber found that two particular “aging body reminders” predicted an older subjective age; everyday body problems (e.g., balance issues, pain, sleep problems, etc.) and body repairs (e.g., reparative surgery). Of the two predictors, everyday body reminders had a stronger effect; each additional everyday body reminder predicted an increase in subjective age of 8 months. This finding points to the relevance of everyday experiences for subjective age, and highlights that more research is needed in identifying the daily context of these reminders.

**Figure 2:**
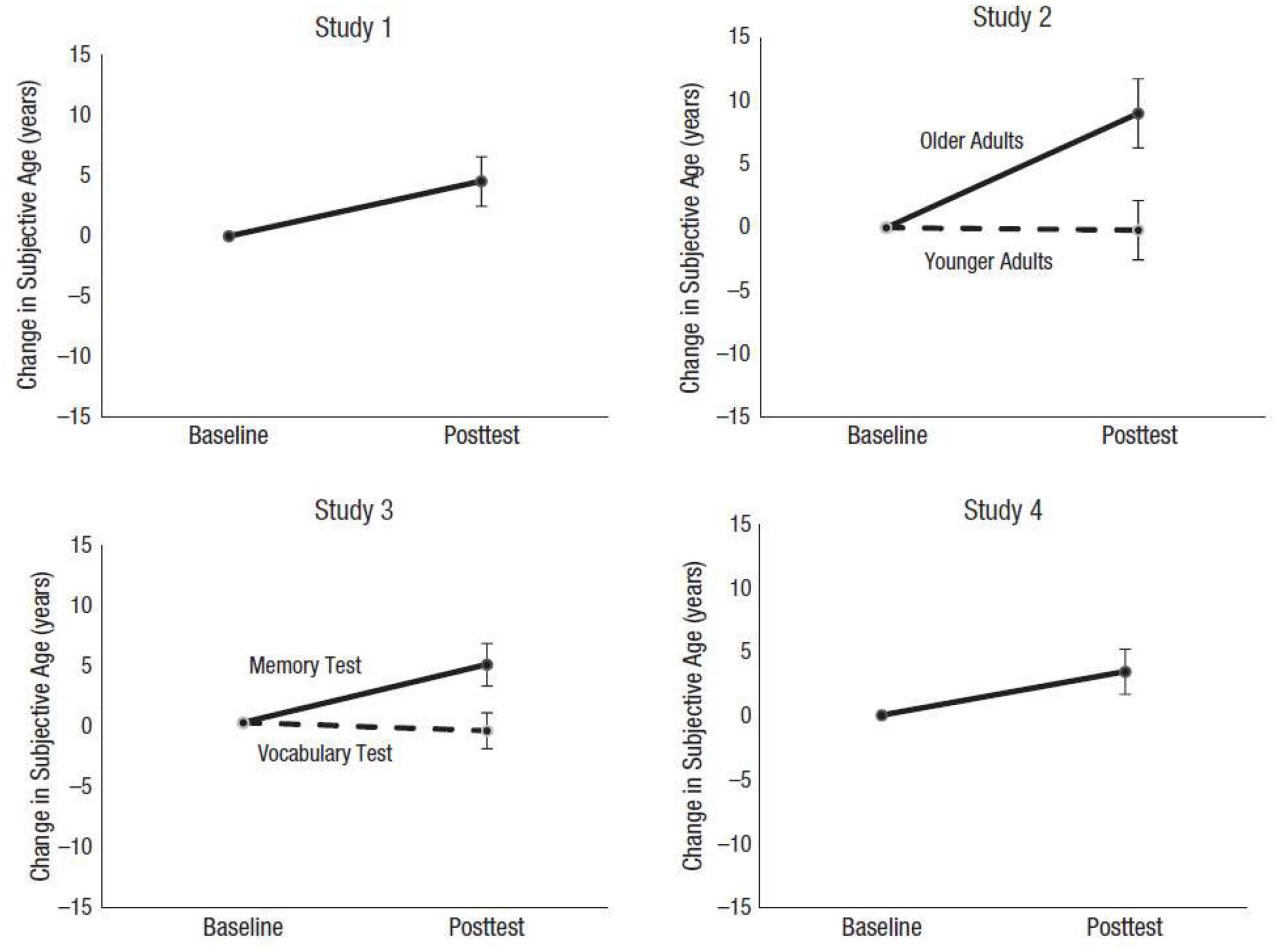
Aging 5 years in 5 min. Results from Hughes et al. (50). Baseline subjective age was centered at zero for each study, and post-test subjective age is plotted as the difference from baseline after a memory test or vocabulary test. In Study 4, older adults were given instructions for a memory test, but reported their subjective age before taking the test. Error bars represent ±1 SE.

Related work in the laboratory has examined how cognitive assessments impact subjective age. Older adults who take a memory test, but not a vocabulary test, have reported feeling older relative to their pre-test subjective age (50). Over a series of studies, older adults felt on average 5 years older after taking a memory test (see Figure 2). Younger adults did not demonstrate this effect. Older adults who took a vocabulary task did not report an older subjective age either, suggesting that there was something unique to memory tests that induced an older felt age in older adults. As with grip strength, older adults taking a memory test may hold certain expectations. Memory is expected to decline with old age, and participants may have concerns about performing poorly. Although no explicit feedback was provided to participants in this study, participants may have implicitly recognized that they had forgotten some words through metacognitive monitoring processes. Failure to recall some of the studied words may have highlighted beliefs and stereotypes about aging and thereby induced an older felt age. Vocabulary tests are not burdened with the same age-related expectations of decline, and thus did not trigger an older subjective age. Taken together, these studies demonstrate that subjective age is dynamic and pliable, and not a static variable.

Subjective age is impacted by one's beliefs and expectations about aging. For example, people generally anticipate that cognitive ability, namely memory, will suffer substantial declines with age (51–53). Such beliefs are rooted in societal expectations of aging and may not reflect a person's actual ability. However, these inaccuracies may still have a negative impact on well-being and influence everyday behavior (51). Without feedback on their performance, older adults may assume their memory is declining, or poor relative to others their age, making them feel subjectively older.

## The Problem With a Unitary Subjective Age

Kastenbaum et al. (6) originally defined subjective age using two domains, concluding that the two factors “Feel Age” and “Look Age” were separate aspects of the concept “Personal Age.” This separation suggests an important distinction between internal feelings of age, and external, observable factors. A third concept of “Ideal Age” suggested that subjective age may be defensive, that older adults report feeling younger because it is more desirable (1). Regardless, subjective age was treated as a single factor in much empirical research, measured only by asking people “How old do you feel,” until Montepare (7) outlined a multidimensional construction of subjective age. In this view, subjective age is defined as a set of values describing several dimensions of felt age. It has been suggested that the adherence to a unitary measure of subjective age, despite early calls for a multidimensional conceptualization, may have caused a period of disinterest in subjective age research (17).

Montepare (7) separated subjective age into Psychological Age (also classified as Cognitive Age), Physical Age, and Social Age, three important life domains for older adults. This view emphasized the importance and distinction of these life domains for subjective age; an individual can feel younger mentally, but older physically. This perspective also suggests that subjective age depends on one's situational context. That is, a person might feel young when they successfully remember a grocery list (a cognitive task) but feel much older when they try to carry the heavy groceries out to the car (a physical challenge).

Others have suggested that subjective age can vary by life domain, which they define as: family; friends or social relations; leisure; personality; finances; work; and physical, mental fitness, health, and appearance (22). Still more work included the concepts of “do-age” and “interest-age” (54). In these definitions, it is the context that is important for one's subjective age, as opposed to individual characteristics.

The distinction between “awareness of age-related change” (8, 17) and “attitudes toward one's own aging” (55) also speaks to the multidimensionality of subjective age. Awareness of age-related change describes a person's experience and recognition of their aging process. Diehl et al. (17) have argued that research on subjective age has been lacking in attention to individual experiences. Relatedly, attitudes toward one's own aging also reflects whether a person views their aging positively or negatively. Brothers et al. (55) demonstrated that the three concepts are related, yet distinct. Felt age predicted functional health and well-being, but these effects were mediated by awareness; participants who reported an older subjective age also noted more negative age-related changes, which predicted poorer health and well-being. These findings support the idea that subjective age is not a single construct, and that subjective age can capture both positive and negative aspects of the aging process. Although the authors propose that awareness of change is a facet of subjective age, we argue here that this is consistent with the idea that experiences and contexts are vital to the construction of subjective age. That is, the self-awareness of age-related change is driven by a person's experiences, and it is these experiences that inform their subjective age. Our contextual model further describes that these experiences and feelings of subjective age feed into decisions about everyday activities, which ultimately lead to health outcomes.

## Stereotypes of Aging

Aging stereotypes reflect a set of socially shared beliefs about how people age (10). Situations that impact subjective age in experimental work often rely on people's expectations of the aging process (i.e., declines in vision, strength, or memory). Given their reliance on interpretations of age-relevant experiences, it is unsurprising that age-based stereotypes have also been connected to subjective age (56). Several studies that have demonstrated changes in subjective age take advantage of aging stereotypes, such as failing eyesight, strength, or memory. It has been proposed that people feel subjectively older when they experience age-based stereotype threat. In contrast, Hughes et al. (50) found that a non-stereotyped cognitive task (i.e., vocabulary) did not make older adults feel older. Experiences in areas that do not involve stereotypes may not impact subjective age, whereas challenging and stereotyped task experiences (e.g., memory) are more likely to. Although there is suggestive evidence that aging stereotypes are tied to subjective age (for example, visual disfluency making people feel older, physical hand grip making people feel younger, memory test making people feel older) there is little direct evidence of this link.

The evidence on the link between subjective age and aging stereotypes is equivocal, and a direct examination of stereotype threat did not find evidence for a link to subjective age (57). Stereotypes of aging may be more relevant to some people than to others. Levy's Stereotype Embodiment Theory (25) postulated that stereotypes gain salience when certain events make them personally relevant to an individual. Stereotypes are learned and internalized throughout the lifespan and may even operate subconsciously until they become salient. The equivocal findings on stereotypes and subjective age may be attributed to the level of personal salience a stereotype holds for an individual. Furthermore, stereotype embodiment may work through multiple pathways, psychological, behavioral, and physiological. The multidimensional nature of stereotypes further stresses the importance of measuring subjective age in a multidimensional way that can align these pathways with the appropriate subjective experience of aging.

Why then do age-stereotyped tasks exert such a robust effect on subjective age? The answer could lie not in the stereotypes themselves, but instead in the types of thoughts older adults have within the situational context. For instance, instead of feeling threatened by a stereotype, and as a consequence feeling older, perhaps a stereotyped situation about cognition and aging would make an older adult think more about their own mental processes. They might examine their performance or compare their memory to that of their same-age peers. This metacognitive monitoring and reflection process can be specific to a task or situation but may also have a broader impact. If such thoughts lead one to assume their memory is failing, this may cause them to feel older. If we do not account for the reflective thoughts that result from experiences, variation across situations and individuals might obscure meaningful patterns in the link between stereotypes and subjective age.

It is reasonable to expect that the way in which a person's culture views and treats older adults would have an impact on how old they feel. Cross-culture examinations of subjective age over 18 countries found the majority of people report a younger subjective age (58). However, the discrepancy between subjective age and chronological age has been found to be larger in American samples when compared to German samples (59). These discrepancies might be even further explained by looking at life domains. American samples have shown a stronger link between health and subjective age than German and Chinese samples, while links between social structures and subjective age were stronger in the German and Chinese samples than in American samples (60). Although there are typically more similarities than differences in subjective age across cultures, older adults feeling younger than their actual age may not be universal. In a sample of older adults from the country of Dakar, people reported only feeling about 2.6 years younger than their chronological age (61). Although most people in this sample reported feeling close to their actual age, better health predicted a younger subjective age, just as in samples from other cultures. These studies suggest that although culture context is an important factor in determining one's subjective age over the lifespan, a closer look is needed at how different everyday experiences can influence a person's felt age.

## Cumulative Effects of Chronic Situations

Older adults are likely to experience certain events regularly. Some of these events may seem benign, but repeated exposures to negative or stressful events can have a cumulative effect on subjective age. One approach to researching the impact of daily experiences is the use of daily diaries. This approach considers multiple occasions of measurement, as opposed to a single episode. In these studies, older adults retrospectively record their thoughts and events at the end of each day, typically for 1 or 2 weeks. The daily diary approach embraces the idea that subjective age can fluctuate and be influenced in important ways from day-to-day.

Daily diary studies have provided critical evidence that personal experiences contribute to subjective age. One of the first daily diary studies to demonstrate day-to-day fluctuations in subjective age also found that these fluctuations were not explained by the passage of time; for example, participants did not feel older at the end of the study compared to baseline. Instead the fluctuations were related to contextual variables that also occurred day-to-day (62). These situational influences that predicted feeling older on a given day included more daily health problems, more daily stressors, and negative affect.

**Figure 3:**
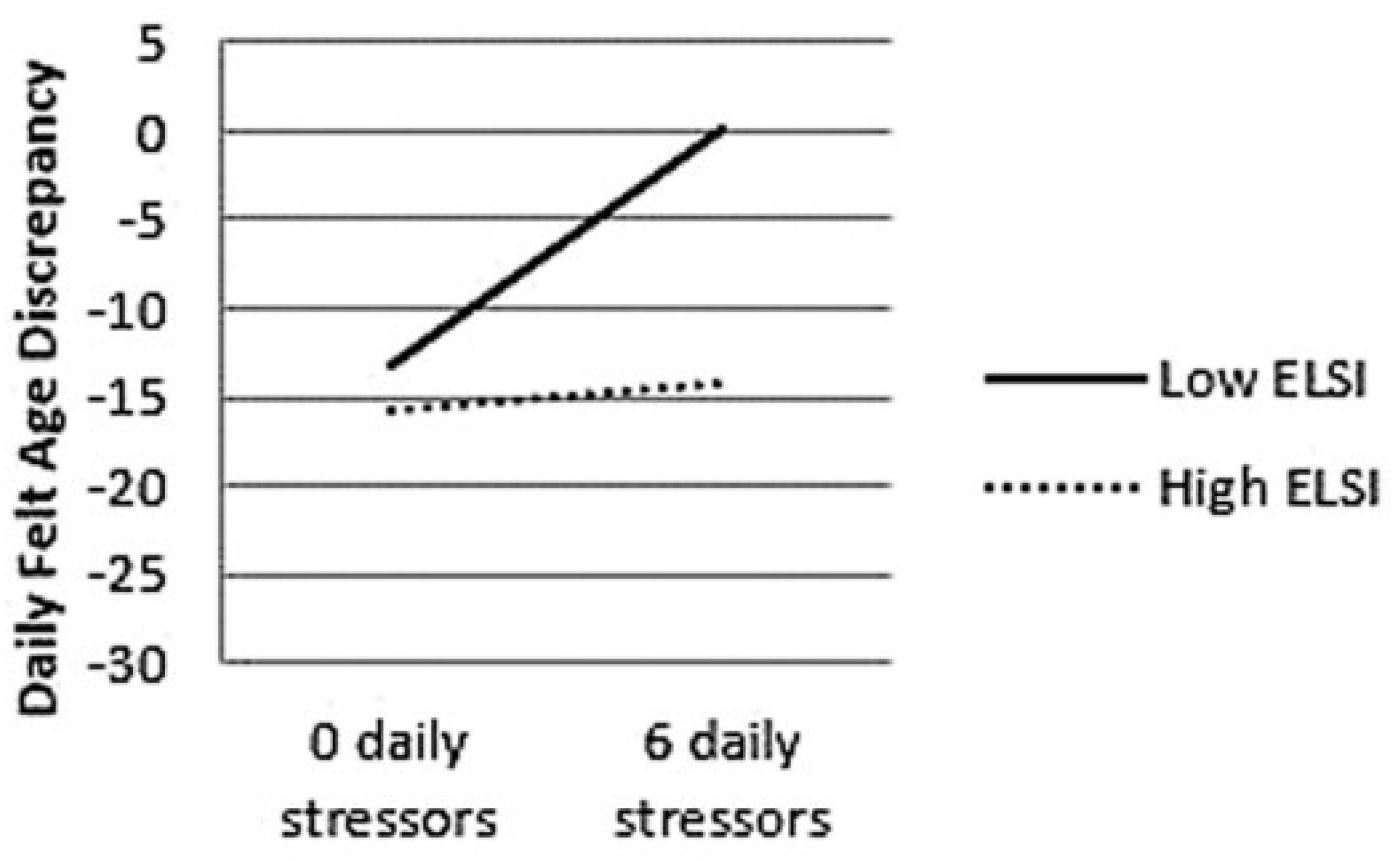
Effects of daily and major life stressors on subjective age. Results from Bellingtier et al. (63) show that those with fewer major life stressors (measured by the Elder Life Stress Inventory, ELSI) show greater increases in daily felt age in response to a stressor compared to those with higher levels of life stressors.

Another study found that daily stress and major life-stressors have a profound impact on felt age, with each daily stressor adding about 2.5 additional years to felt age (63). This effect was mediated by negative affect, again highlighting the relevance of how a person experiences events in constructing their subjective age. Additionally, the effect of daily life stressors on subjective age was smaller for individuals who experienced a major life-event stressor (e.g., death of a close friend, deterioration in health) in the past year (Figure 3). These major life events may have served as a contextual reference point, making daily stressors feel less impactful on felt age.

Although only a few studies have looked at situational changes in subjective age, they have demonstrated that these contexts are important for subjective age. One potential limitation of this work is that it relies on retrospective reports. Therefore, these studies cannot separate the immediate impact of experiences on subjective age from their cumulative and perhaps more general effect. These studies ask individuals to respond once, at the end of the day, to the question “How old do you feel today?” In order to better understand how these specific everyday experiences impact subjective age, future work must assess subjective age as situational changes occur.

We believe that people use subjective age as a form of self-knowledge to influence their decision-making. Because subjective age represents a self-assessment, and because these assessments can be impacted by factors like cognitive challenges (50) or stress (63), it is important that we understand how subjective age behaves moment-to-moment. In the section that follows, we discuss methodological issues in the study of subjective age and suggest advancements that will enable a more thorough study of subjective age within the context of life experiences.

## Measurement of Subjective Age

Just as research on subjective age has evolved, so has the way we ask participants to report their felt age. In recent work, researchers ask people what age they feel by requesting a specific felt age. When measuring subjective age repeatedly over the course of a single study, this may anchor participants to a given value and complicate detection of fluctuations in subjective age. For this reason, Hughes et al. (50), Geraci et al. (64), and Marquet et al. (57) devised an unmarked slider response scale. This scale can be used in quick succession, in as little as 5 min, without the risk of participants simply repeating their original answer, and so has proven useful in detecting meaningful subjective age fluctuations that occur in moment-to-moment contexts.

### Single or Multiple Occasions

Typical studies of subjective age use a single occasion measurement that captures felt age for only one episode. This approach ignores the possibility that subjective age can change, depending on the situation. However, there is a relatively small body of longitudinal research that does incorporate multiple measurements of subjective age, albeit usually as single occasion measurements that are many years apart. These longitudinal studies have shown relationships between subjective age and personality (65), cognitive functioning (14), and social comparisons of health and cognition (66) over two measurements roughly 10 years apart.

The daily diary methods described above have taken a step in this direction. By repeatedly measuring subjective age daily over the course of a week, we can start to see how subjective age changes over shorter time intervals. The daily diary method also addresses another issue in the subjective age literature: subjective age tends to be measured in a laboratory setting. By allowing participants to report their subjective age at home in a daily diary, it moves us closer to assessing how subjective age operates in real life. However, daily diary studies do not address another issue of subjective age research, the retrospective nature of the question. People were still asked to think about their felt age at the end of the day, which requires them to reconstruct the day's events in their head. To fully understand how subjective age operates, we argue that we need to “catch subjective age in the moment.” Below we describe a promising approach toward that aim.

### Experience Sampling

As described earlier, laboratory experiences can have an immediate and substantial impact on subjective age (43, 48, 50). A critical extension of this work is the study of how experiences impact subjective age in everyday life. To tackle this question, and others, we need to devise methods that accurately measure subjective age in real time in the real world. One promising strategy is the use of Experience Sampling Methodology (ESM). ESM studies of subjective age would ask participants how old they feel at random intervals throughout the day and collect information about the individual's situational context and perceptions. Such research will provide us with key insight in how daily experiences in varying contexts impact subjective age.

A substantial body of research has successfully explored other aspects of older adults' *in vivo* experiences using ESM [e.g., (67–71)]. For example, Droit-Volet and Wearden (67) studied time perception by giving older and younger adults a smartphone which produced 8 probes a day for 5 days. At the sound of a beep, participants were asked to answer a 12-item questionnaire, which included questions about what the person was doing, who they were with, how they felt, and how they perceived the passage of time. This study found no difference in the perception of time between older and younger adults but found that affective states did influence the perception of time at any age. Another study by Scott et al. (69) studied *in vivo* stress by probing a sample of adults between the ages of 20 and 81 five times a day for 10 days. Their results found that older adults were less likely to experience an increase in negative or a decrease in positive affect immediately after a stressor compared to younger adults. There were no age differences for the impact of stress that occurred more than 3 h prior, suggesting that chronic stress is important for the emotional experience of everyday life.

These studies demonstrate not only the feasibility of using ESM in an older population, but also that everyday experiences and contexts can be impactful. Such research can provide critical insight into the similarities and differences in everyday life for younger and older adults. Given the prevalence of smart devices in society, and the increasing number of older adults that use and rely on these devices, we should take advantage of the technological and societal advances that can facilitate this approach. There still remain important questions in how to implement these approaches. For example, what would be the optimal schedule of assessment (e.g., event vs. interval scheduling). We should also consider that all events are not equally relevant for subjective age; some events will be highly age-relevant, while others may not be relevant to age at all. Integrating these methods would answer an earlier call to conduct ESM studies on subjective age (72), allow researchers to take advantage of the high within-person power of these designs, and uncover how the immediate context impacts subjective age, as described in the model presented here.

## Lifestyle Choices

The final step in our contextual model connects subjective age with health and well-being through momentary lifestyle choices. There is a large body of research demonstrating that subjective age predicts health outcomes [e.g., (12, 13)], but the underlying mechanisms are still unclear. Here too, we can fill these gaps with ecological assessments. In our contextual model, we propose that subjective age affects the types of lifestyle choices a person makes, which then ultimately affect health. Supporting this view, Levy and Myers (73) have shown that people with more positive self-perceptions of aging engage in more preventive health behaviors, such as maintain a healthy diet, exercising, and complying with medications. Exercise interventions paired with a component that emphasized positive views of aging have been effective at changing attitudes toward older adults and increasing physical activity in older adults (74). The change in aging attitudes predicted the increase in physical activity and was more effective than exercise alone.

In two longitudinal datasets from the Health and Retirement Study and the National Health and Aging Trends Study, a younger subjective age was associated with a faster walking speed at baseline and follow-up 2 to 4 years later (65), as well as less decline in walking speed. True to the multidimensional perspective of subjective age, domain-specific physical subjective age also predicts physical activity (75). Younger physical subjective age predicted a higher level of physical activity over a 4 week span, and more intended physically activity. Other work has also not only found that subjective age predicts physical activity intention, but that this relationship is partially mediated by exercise self-efficacy (76). People may also engage in certain leisure behaviors if they feel younger, such as traveling for vacations (77). Healthy lifestyle choices can also explain subjective age's relationship to cognition in later life (14). A large body of recent work demonstrates that engaging in light fitness training preserves cognitive function in older adults [e.g., (78)]. Connecting this research to our proposed model, we believe that a younger subjective age promotes more confidence in a person's physical ability, which promotes healthy lifestyle behaviors. These healthy lifestyle behaviors can explain the link between subjective age and health outcomes and present the best opportunity for targeted interventions. Our contextual model adds to work in this area by providing a framework for these relationships and their underlying mechanisms.

## Conclusions

Research has demonstrated that one's felt age is an important factor in their well-being. A younger subjective age predicts beneficial health outcomes, although some have warned that denying an aspect of your identity may have lasting psychological consequences [e.g., (8)]. It seems that subjective age contains self-reflective information with a more accurate assessment of a person's aging process than their chronological age, and that this information is shaped by that individual's daily life experiences. We argue that the next step in subjective age research should be to focus on how personal experiences and reflections contribute to one's felt age. Although other researchers have pointed to the relevance of personal experiences in subjective age (17, 56, 79), these approaches are often framed on a macro-level, either taking a life-span approach or a societal view of aging stereotypes and stigma. We propose a more fine-grained look at how subjective age functions in everyday life.

Our contextual model of subjective age (Figure 1) illustrates that we must first discriminate the links between situational influences and subjective age in order to better understand the associations between subjective age, lifestyle choices, and health outcomes. In this paper, we have outlined research showing that subjective age should be considered multidimensional, and that these dimensions of subjective age can be influenced by different contexts. As we move from one context to another, our subjective age is likely to fluctuate as well. We argue that the study of subjective age as it fluctuates in response to life experiences can help us refine the final links in our model, by connecting subjective age to behaviors which ultimately impact health and well-being in a manner similar to the behavioral pathway in stereotype embodiment theory (25). It is important to note that the model is likely to be cyclical, and that health forms a connection with situational context, as well as subjective age, such that poor well-being may lead to more situations that make one feel older (e.g., visiting a doctor's office, or experiencing greater challenge with everyday activities).

Future research should closely investigate how daily experiences, such as physical and cognitive challenges or successes, impact subjective age on a momentary basis. Treating subjective age and life contexts as multidimensional will expand our knowledge of subjective age and allow us to pinpoint what factors contribute most to a person's overall felt age. We must also consider how individuals perceive and respond to life events. By uncovering the discrete role of daily experiences in situational contexts, we will better understand the nature of self-knowledge implicit in a person's subjective age.

A key feature of this approach is to uncover the factors that people rely on to construct their subjective age. This understanding will help us predict the specific *in vivo* experiences a person faces that cause subjective age to fluctuate. At the center of our contextual model of subjective age is a person using their daily experiences to make minor and major decisions. Going forward, it is critical to know how experience and context alters subjective age, and how subjective age influences these everyday lifestyle choices.

## Author Contributions

All authors listed have made a substantial, direct and intellectual contribution to the work, and approved it for publication.

## Conflict of Interest

The authors declare that the research was conducted in the absence of any commercial or financial relationships that could be construed as a potential conflict of interest.
